# Genome-wide analysis of *RopGEF* gene family to identify genes contributing to pollen tube growth in rice (*Oryza sativa*)

**DOI:** 10.1186/s12870-020-2298-5

**Published:** 2020-03-04

**Authors:** Eui-Jung Kim, Sung-Wook Park, Woo-Jong Hong, Jeniffer Silva, Wanqi Liang, Dabing Zhang, Ki-Hong Jung, Yu-Jin Kim

**Affiliations:** 1 Graduate School of Biotechnology and Crop Biotech Institute, Kyung Hee University, Yongin, 17104 South Korea; 2 Joint International Research Laboratory of Metabolic and Developmental Sciences, State Key Laboratory of Hybrid Rice, School of Life Sciences and Biotechnology, Shanghai Jiao Tong University–University of Adelaide Joint Centre for Agriculture and Health, Shanghai Jiao Tong University, Shanghai, China

**Keywords:** RopGEF, ROP/Rac, Pollen, *Oryza sativa*, Gene family

## Abstract

**Background:**

In plants, the key roles played by RopGEF-mediated ROP signaling in diverse processes, including polar tip growth, have been identified. Despite their important roles in reproduction, a comprehensive analysis of RopGEF members has not yet been performed in rice (*Oryza sativa*). To determine whether RopGEF regulators are involved in rice pollen tube growth, we performed genome-wide analysis of this family in rice.

**Results:**

Phylogenomic and meta-expression analysis of eleven RopGEFs in rice showed that four genes were preferentially expressed in mature pollen. These four genes contain the plant-specific Rop nucleotide exchanger (PRONE) domain and possible phosphorylated residues, suggesting a conserved role in polar tip growth with *Arabidopsis thaliana*. In subcellular localization analysis of the four *RopGEFs* through tobacco (*Nicotiana benthamiana*) infiltration, four proteins were predominantly identified in plasma membrane. Moreover, double mutants of RopGEF2/8 exhibited reduced pollen germination, causing partial male sterility. These genes possess unique cis-acting elements in their promoters compared with the other RopGEF genes.

**Conclusions:**

In this study, four RopGEF genes were identified as pollen-specific gene in eleven members of rice, and the expression pattern, promoter analysis, and evolutionary relationship of the RopGEF family were studied compared with Arabidopsis. Our study indicated that four RopGEF genes might function during pollen germination in distinct subcellular localization. Our study could provide valuable information on the functional study of RopGEF in rice.

## Background

Rho-type GTPases of plants (ROP), also known as RACs, are a plant-specific subfamily of Rho small GTP-binding proteins [1] and participate in diverse signal transduction processes, including disease resistance, pollen tube growth, root hair development, reactive oxygen species (ROS) production, cell wall patterning, and hormone responses [2]. Similar to Rho family proteins in other eukaryotic cells, ROPs regulate endocytosis and exocytosis through cytoskeleton organization and intracellular kinase cascades through activation of NADPH oxidase, processes that are key for cell polar growth in plants [3–6]. Activation and inactivation of ROP, by change in its conformation following GTP binding and hydrolysis (GDP-binding form), functions as a molecular switch in signal transduction [6, 7]. Conversion from the GDP and GTP form of Rho GTPases is catalyzed by guanine nucleotide exchange factors (GEFs). Plant-specific RopGEFs spatiotemporally regulate the activity of ROPs/Racs through the catalytic plant-specific ROP nucleotide exchanger (PRONE) domain [8].

Although few studies of *RopGEF*s have been carried out in other plant species, recent studies in the model plant Arabidopsis support their roles in various plant development processes as well as in defense. Arabidopsis has 14 *RopGEFs* in its genome, with a high degree of sequence similarity [8, 9]. Functional studies showed that AtRopGEF1 and AtRopGEF4 specifically regulate ROP11 in abscisic acid (ABA)-mediated stomatal closure [10], and that AtRopGEF1 plays a role in controlling lateral root growth [11] and polar auxin transport to achieve cell polarity during early plant development [12]. Among them, seven members are specifically or highly expressed in pollen tubes and function redundantly [8, 13]. Pollen-specific RopGEFs have conserved C-termini, which function by auto-inhibition of the PRONE domain [13]. Recent findings suggest that pollen-specific receptor-like kinases (PRKs) transduce ROP signaling via phosphorylation of RopGEFs at the C-terminus to regulate polar pollen tube growth [13–17]. Germinated pollen tube enters the female gametophyte and delivers sperm cells to egg cells and polar central cells, which perform double fertilization in flowering plants [18]. When some ligands are received by receptor-like kinases (RLK), RopGEFs are activated and convert GDP to GTP, which binds to ROP [19]. AtPRK2 promotes ROP1 via phosphorylation of RopGEFs in the control of polarized pollen tube growth [16]. Interaction of AtPRK6 with AtRopGEF8 and AtRopGEF12 plays essential roles in polarized growth of pollen tubes [17]. Another RLK, FERONIA, belonging to the *Catharanthus* receptor-like kinase (CrRLK) family, was shown to function as an upstream regulator of RopGEFs, mediating auxin efflux on root hair growth in Arabidopsis [20–22].

In rice (*Oryza sativa*), among the first 12 reported genes, 11 *OsRopGEFs* encoded full-length proteins [23]. *OsRopGEF*s were shown to play a role in the activation of *OsRac1* in the disease-resistance response [24]. *OsRac1* negatively regulates cell death and innate immunity by production of ROS [25, 26]. It was shown that *OsRac1* activates plant NADPH oxidase, known as OsRbohB (respiratory burst oxidase homolog), by direct interaction [27]. In addition, *OsRopGEF10* activates the development of small cuticular papillae on leaf surfaces [28], whereas *OsRopGEF7B* is involved in regulating floral organ development [29]. Compared with Arabidopsis, the molecular function of rice RopGEF members in response to plant developmental cues is poorly understood.

To reveal whether roles in polar tip growth in rice exist for the conserved RopGEFs, we investigated in the present study the phylogenetic relationship of rice and Arabidopsis RopGEF proteins, analyzing the PRONE domain and characterizing the amino acid residues. The molecular mechanism on rice pollen tube growth is less known, in spite of its importance on gamete transmission for successful reproduction [30]. We identified four pollen-preferred *OsRopGEF* genes through meta-expression analysis using our pollen/anther database, followed by confirmation by qRT-PCR. In addition, we performed subcellular localization of each RopGEF through tobacco infiltration and *cis*-acting element (CRE) analysis of the four pollen-preferred *OsRopGEF* genes, revealing a conserved CRE. Finally, we generated and analyzed single and multiple knock-out mutants for the functional studies.

## Results

### Expression profiling analysis of *RopGEF* genes

**Fig. 1:**
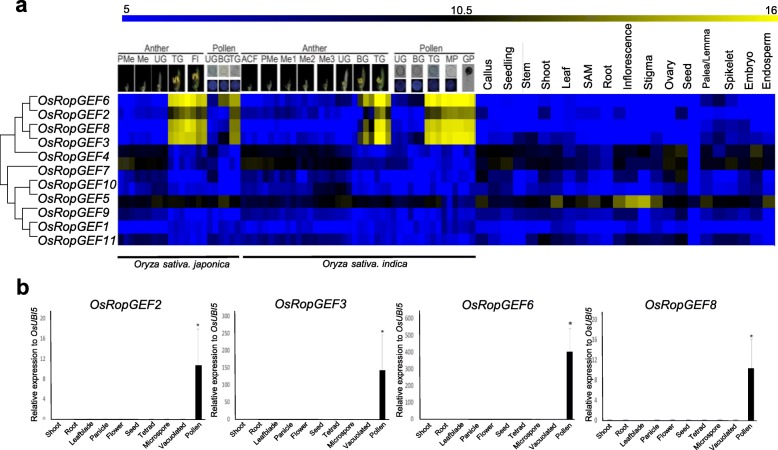
Meta-expression analysis and genome-wide identification of *OsRopGEF* and validation of meta-expression patterns of pollen-specific *OsRopGEF*s using qPCR. **a** Heatmap expression and phylogenetic analysis of 11 *OsRopGEF* genes revealed four genes are specifically expressed in MP. 22 microarray data for *indica* rice including five stages for anthers and three for pollen and 42 microarray data for *japonica* rice including eight stages for anthers and five for pollen were used. ACF, archesporial cell-forming stage; BG, bi-cellular gametophyte stage; Fl, flowering stage; GP, germinated pollen; Me, meiotic stage; Me1, meiotic leptotene stage; Me2, meiotic zygotene-pachytene stage; Me3, meiotic diplotene-tetrad stage; MP, mature pollen stage; PMe, pre-meiosis; TG, tricellular pollen stage; UG, uni-cellular gametophyte stage. Yellow color in heatmap indicates high level of expression; dark blue, low expression. Numeric values indicate an average of the normalized log2 intensity of microarray data. **b** Expression of pollen-preferred *OsRopGEF* genes was analyzed by qPCR in various tissues of rice; the anther of tetrad, microspore, vacuolated pollen stage and mature pollen grains were used. Rice ubiquitin 5 (*OsUbi5*, LOC_Os01g22490) was used as an internal control. Y-axis, expression level relative to *OsUbi5*; X-axis, samples used for analyses. Error bars represent the standard errors of three biological replicates. Significant differences are indicated by asterisks, *, *p*-value < 0.01. Data were analyzed by employing one-way ANOVA with repeated measures, using Tukey’s pairwise comparison test

To identify the pollen-preferred genes of rice, we examined a publicly available Affymetrix rice microarray data [31]. Expression patterns conserved between *indica* and *japonica* rice varieties at various developmental stages of anthers and pollen, and anatomical meta-expression data from our established database, Rice Anther Pollen Expression Database (RAPED) (http://ricephylogenomics-khu.org/RAPED/home.php), were analyzed. We then constructed a phylogenic tree by aligning the protein sequences of all *OsRopGEFs*. Meta-expression data in the context of the phylogenetic tree were combined to check the function of *OsRopGEF*s with respect to anther and pollen development, compared with other tissues/organs (Fig. 1a). As a result, the rice *RopGEF* gene family can be divided into two subfamilies, one group to which *OsRopGEF2, 3, 4, 6, and 8* belong, and the other to which the remaining six genes belong, namely *OsRopGEF1, 5, 7, 9, 10,* and *11*.

In addition, we identified that *OsRopGEF2, OsRopGEF3, OsRopGEF6*, and *OsRopGEF8* showed selective expression at the stage of late pollen development, i.e., at stages of tricellular pollen grains, mature pollen (MP), and germinated pollen (GP). These four genes were used for further analyzes. Since *OsRopGEF3* was also highly expressed in root hairs, we predicted that this gene could affect tip growth (Additional file 1). Genes *OsRopGEF4* and *OsRopGEF7* showed some expression in the early stages of anther development, although their expression levels decreased during the later stages. Furthermore, because expression is also detected in tissues other than anther and pollen, these two genes cannot be considered to be pollen-preferred expressed genes. In addition, *OsRopGEF5* showed weak expression in *japonica* rice anthers and other tissues, showing no preferred expression in anthers and pollen.

Next, to verify the meta-expression data, we performed quantitative real-time PCR (qPCR) using ten tissues: shoot, root, leaf blade, panicle, flower, seed, tetrad microspore, young microspore, vacuolated pollen, and MP (Fig. 1b). By sampling anthers at various developmental stages, from pollen mother cell to MP and GP, we tried to determine at which stage the target genes were being expressed. As a result, *OsRopGEF2, OsRopGEF3, OsRopGEF6*, and*OsRopGEF8* were shown to be highly expressed in MP. *OsRopGEF6* showed the highest level of expression in MP, and *OsRopGEF2* and *OsRopGEF8* showed similar expression levels in MP. These native pollen-preferred *OsRopGEFs* were generally not expressed in other tissues, similar to meta-expression profiles using microarray data. Especially in rice, gene expression profiles of mature and germinated pollen were highly correlated [31]. Therefore, these four genes may play key roles in pollen tube growth and other processes that occur after pollen maturation.

### Comparative analysis of the conserved domains in RopGEF genomic and protein sequences between rice and Arabidopsis

**Fig. 2:**
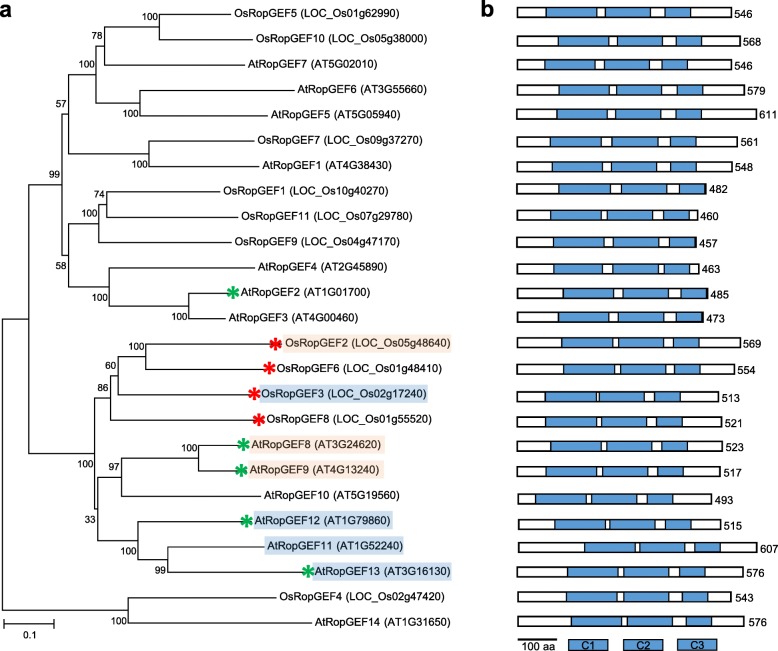
Phylogenetic analysis of RopGEF family from *Oryza sativa* and *Arabidopsis thaliana*. **a** The phylogenetic tree was constructed by annotating the protein sequences for each gene by using MEGA7. Protein sequence alignment was performed by ClustalW and phylogeny by Neighbor-joining methods, with the reliability being estimated by the 1000 times bootstrap test. The number between the trees represents the bootstrap value. Os, *Oryza sativa*; At, *Arabidopsis thaliana*. Red and green asterisks indicate *RopGEF* genes with high expression in pollen in *O. sativa* (in this study) and *Arabidopsis thaliana* [8], respectively. Red and blue boxes indicate the orthologous relation between rice and Arabidopsis *RopGEF* genes indicated in RGAP. **b** A scheme of protein structure. We arranged the sequence of the protein domain scheme to be the same as the gene sequence of the phylogenetic tree of A. Length of each RopGEF protein is shown on the right and its unit is aa (amino acid). C1, C2, and C3 (blue boxes) indicate position and length of the conserved PRONE subdomain of the RopGEF family

Genes *OsRopGEF2, OsRopGEF3, OsRopGEF6*, and *OsRopGEF8* encode proteins with similar amino acid sequences. *RopGEF*s are considered to be a novel gene family with a unique structure containing the PRONE catalytic domain that is exclusively found in the plant *RopGEF* gene family [9]. The PRONE domain is known to be required for RopGEFs to convert GDP to GTP. The PRONE domain of RopGEFs is highly conserved in all OsRopGEFs. More interestingly, the pollen-preferred OsRopGEFs were found to retain the C-terminus region after the PRONE domain (Fig. 2b), whereas the pollen-non-preferred *OsRopGEF1*, *OsRopGEF9,* and *OsRopGEF11* lack this C-terminus.

For the comparative analysis of *RopGEF* gene families*,* we collected the protein sequences of 14 *AtRopGEFs* and 11 *OsRopGEFs* and constructed a neighbor-joining tree (Fig. 2a). Four *OsRopGEF* genes showing pollen-preferred expression were clustered together with *AtRopGEF8* and *9*, which also exhibited pollen-preferred expression. In the Arabidopsis *RopGEF* family, the expression patterns revealed by qPCR were not consistent with those from previous reports: *AtRopGEF1, 8, 9, 12, 14* were detected in pollen tissues [8] and *AtRopGEF8, 9, 10, 11,* and *13* showed pollen-preferred expression, respectively, and, of these, the latter data corresponded more closely with the transcriptome data in Genevestigator (Additional file 2). According to Rice Genome Annotation Project (RGAP, http://rice.plantbiology.msu.edu/), the orthologs for *OsRopGEF2* are *AtRopGEF8* and *AtRopGEF9,* and those for *OsRopGEF3* are *AtRopGEF11*, *AtRopGEF12*, and *AtRopGEF13*. However, the orthologs for *OsRopGEF6* and *OsRopGEF8* have not been identified. In the phylogenetic tree, rice members with pollen-preferred expression were closely clustered with those in Arabidopsis and the C-terminal amino acid sequences were more similar than those for other members in rice. The invariant serine residue (S510, numbered as in AtRopGEF12) within the C-terminus, which is important for C-terminal inhibition [13] was also located in pollen-preferred *RopGEF* members in rice (Additional file 3), supporting the hypothesis that phosphorylation-regulated GEF activity was conserved.

### Protein structure

**Fig. 3:**
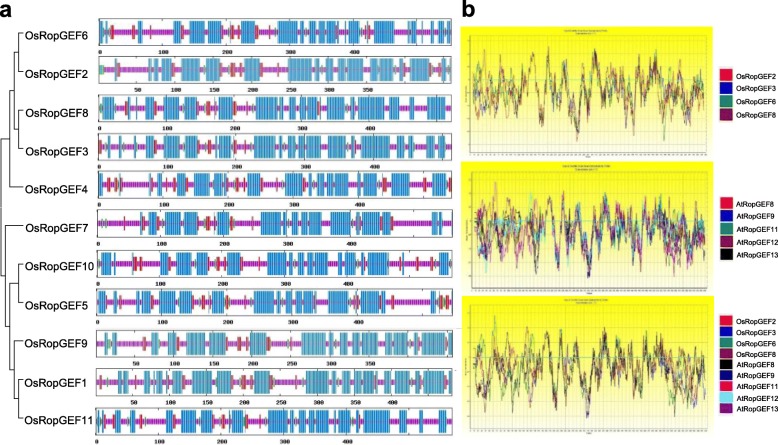
Protein sequence analysis. **a** Secondary structure of RopGEF using the deduced amino acid sequences from OsRopGEFs. Blue lines indicate alpha helices, purple lines indicate random coils, red lines denote extended strands, and green lines represent beta turns. **b** Hydropathy plot analysis. The upper part of the figure is the hydropathy plot of four OsRopGEFs which showed pollen-specific expression, and the central part is a hydropathy plot of five *AtRopGEF* genes that are pollen-specific expressed in the Genevestigator. The last figure is the sum of four OsRopGEF and five AtRopGEF hydropathy plots. Profiles of rice and Arabidopsis RopGEFs showed similarities in the PRONE domain, but not for the N and C-termini

**Fig. 4:**
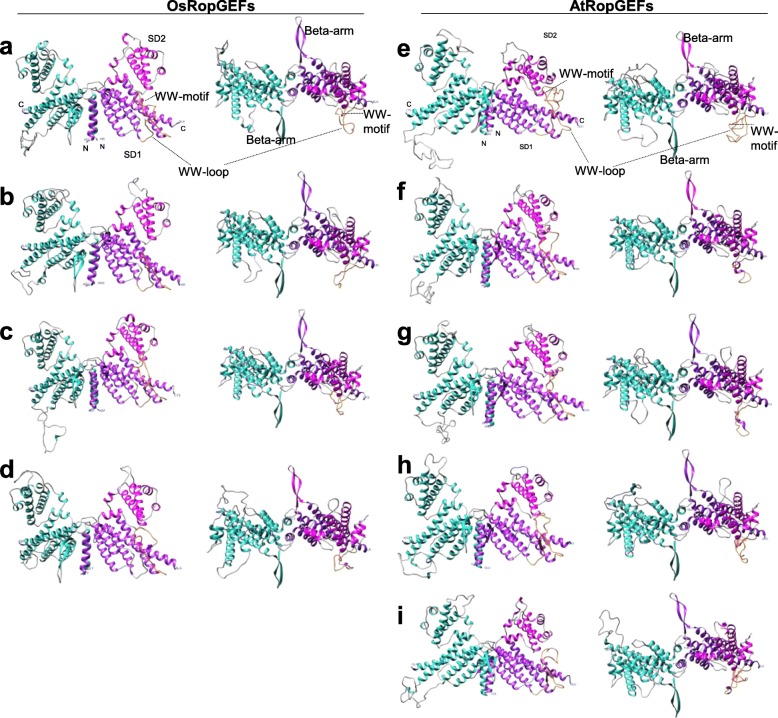
Predicted 3-D structures of *RopGEF* genes in rice and Arabidopsis. **a** *OsRopGEF2*, (**b**) *OsRopGEF3*, (**c**) *OsRopGEF6*, (**d**) *OsRopGEF8*, (**e**) *AtRopGEF2*, (**f**) *AtRopGEF8*, (**g**) *AtRopGEF9*, (**h**) *AtRopGEF12*, and (**i**) *AtRopGEF13*. Comparative representation was performed using the UCSF Chimera package. Coil, left-promoter, subdomain 1, subdomain 2, WW-loop, and WW-motif structures are depicted in gray, sky-blue, purple, magenta, orange, and red, respectively. N- and C- termini are depicted in blue

Protein sequence analysis and hydropathy plot profiling revealed that AtRopGEF and OsRopGEF, both with pollen-preferred expression, had mostly similar amino acid sequences but differed at the N- and C-termini (Fig. 3). The analyzed rice RopGEF 3-D models exhibit a butterfly-shaped three-dimensional structure in which two PRONE protomers dimerize via their N-terminal groups (Fig. 4). Each protomer consisted of two subdomains, with the subdomain 1 containing the WW-loop, which is a common characteristic in Arabidopsis [7] (Table 1). To more precisely characterize the 3-D model structures, we compared the pollen-preferred rice RopGEF 3-D models with those from Arabidopsis. The subdomains differed in the number of alpha helices and the number of residues in the WW-loop between the OsRopGEFs and the AtRopGEFs. Rice and Arabidopsis RopGEF 3-D models displayed trends in the number of α-helices from 14 to 17, and in the number of residues in the WW-loop from 25 to 50.

**Table 1:** Characteristics of 3D predicted model RopGEFs of rice and Arabidopsis

Gene	Number of alpha helices	Number of residues in WW-loop	Subdomain 1 alpha helices	Subdomain 2 alpha helices	Observations
OsRopGEF2	15	40	1–6, 15	7–14	–
OsRopGEF3	14	34	1–6, 14	7–13	–
OsRopGEF6	15	40	1–6, 15	7–14	–
OsRopGEF8	15	25	1–7, 15	8–14	–
AtRopGEF2	14	50	1–6, 14	7–13	–
AtRopGEF8	16	40	1–8, 16	9–15	WW-loop contains two alpha helices (6, 7) and the WW-motif is embedded in alpha-helix 6
AtRopGEF9	15	40	1–7, 15	8–14	WW-loop contains two alpha helices (5, 6)
AtRopGEF12	14	38	1–7, 14	8–13	WW-loop contains two beta-strands
AtRopGEF13	17	40	1–7, 17	8–16	WW-loop contains two beta-strands

OsRopGEFs contained 14 to 15 alpha helices, and 25 to 40 residues in the WW-loop, whereas AtRopGEFs contained 14 to 17 alpha helices, and 38 to 50 residues in the WW-loop. The main difference between OsRopGEFs and the reported AtRopGEF [32] is in the WW-loop, where AtRopGEF displayed α-helices or beta-strands in the WW-loop, whereas OsRopGEFs did not contain α-helices or beta-strands.

### Subcellular localization of four RopGEF proteins

**Fig. 5:**
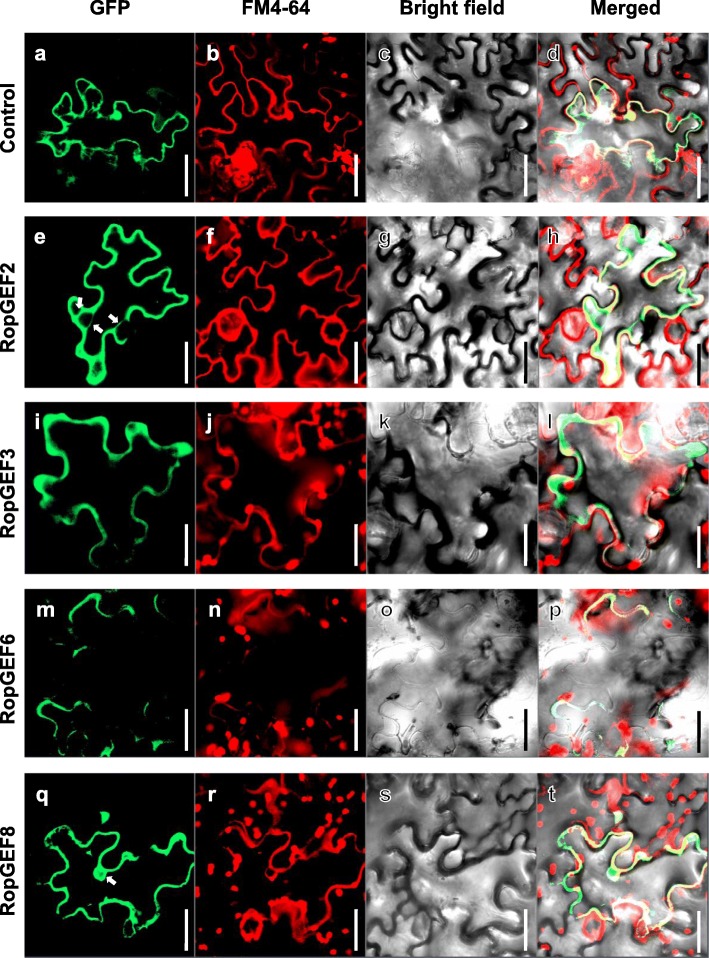
Subcellular localization observation of OsRopGEF proteins in tobacco plants using laser scanning confocal fluorescence microscopy. **a**-**d** Control-GFP, and (**e**-**t**) four pollen-specific expressed OsRopGEF-GFP signals were observed in tobacco epidermal cells. Second panel showed the membrane marker through FM4–64 staining, the third panel showed the bright field image, and last panel showed marge image. The red signal showed membrane, Green signal showed RopGEF localization, and yellow signal showed merged. The white arrow indicates the signal except membrane (RopGEF2-cytoplasm, RopGEF8-Nucleus). Bar = 40 μm

The RopGEF is known to regulate ROP signaling in the plasma membrane (PM). Arabidopsis RopGEF8, RopGEF9, and RopGEF14 were shown to be localized to the apical PM of the pollen tube, and RopGEF1 to the entire PM. On the other hand, RopGEF12 was barely detected in the PM, being present in the cytosol of the pollen tube [8], suggesting that the different localizations of RopGEF in pollen tubes represented differences with respect to multiple regulatory ROP signaling actions. Therefore, we examined the subcellular localization of OsRopGEF members to check whether their spatial locations were the same or different. We introduced RopGEF-GFP fusion proteins controlled by the Cauliflower mosaic virus (CaMV) 35S promoter into the epidermal cells of tobacco leaves, and empty GFP protein was used as a control. The GFP signals of four RopGEF genes were observed in the PM. Further, we used FM4–64 staining as a membrane marker to confirm how the membrane marker and the GFP signal of RopGEF protein correlate (Fig. 5). Control GFP signals were present in the nucleus, membrane and cytosol inside membrane (Fig. 5a-d). Most of the RopGEF protein signals are well merged with the FM4–64 stained RFP signal (Additional file 4). OsRopGEF6 were specifically localized into the PM, consistent with the common RopGEF location (Fig. 5m-p), while the GFP signals of OsRopGEF2, OsRopGEF3 and OsRopGEF8 were predominantly found in the cytosol as well as in the PM (Fig. 5i-l). The GFP signal of OsRopGEF2, located in the cytosol, was seems like associated with the endoplasmic reticulum, rather than the nucleus (Fig. 5e-h) [33], while the OsRopGEF8 signal was associated with the nucleus (Fig. 5q-t) [34]. The results suggest that the four pollen-preferred members of OsRopGEF could carry out unique functions during pollen germination, as reflected by their distinct subcellular locations.

### Promoter analysis of pollen-preferred genes

Up to now, the information about the roles of transcription factors and regulatory motifs in pollen germination in rice has been unclear. Based on the microarray data and the qPCR results, we performed a promoter analysis to identify the *cis*-elements conserved in the promoters of *OsRopGEF*s associated with pollen-preferred expression.

We searched the known *cis*-regulatory elements (CREs) in the promoter based on existing research (Additional file 5). Major pollen-preferred CREs such as POLLEN1LELAT52 (AGAAA), PB Core (CCAC), and GTGANTG10 (GTGA) [35] were identified. In the case of POLLEN1LELAT52, 12 CREs existed in the *OsRopGEF3* promoter and seven CREs existed in the *OsRopGEF8* promoter. In the case of GTGANTG10, 11 CREs existed in the *OsRopGEF8* promoter. In the case of the PB Core, there were five copies in the *OsRopGEF2* promoter, and four copies in each of the *OsRopGEF6* and *OsRopGEF8* promoters. However, in the case of the remaining *OsRopGEF* family genes, there were also many pollen-preferred CREs present in the promoters of genes which exhibited little or no expression in pollen. On average, there were 4.3 copies of POLLEN1LELAT52, 6.4 copies of GTGANTG10, and 1.6 copies of the PB Core in the promoters of the seven *OsRopGEFs* compared with the four highly pollen-preferred genes. We calculated the *p*-values to determine how many of the four highly pollen-expressed *OsRopGEF* genes had significantly more CREs relative to the rest of the *OsRopGEF* genes, but all did not exceed 0.01. Based on these results, we assume that known pollen CREs do not regulate the expression of the four pollen-preferred *OsRopGEF*s.

**Fig. 6:**
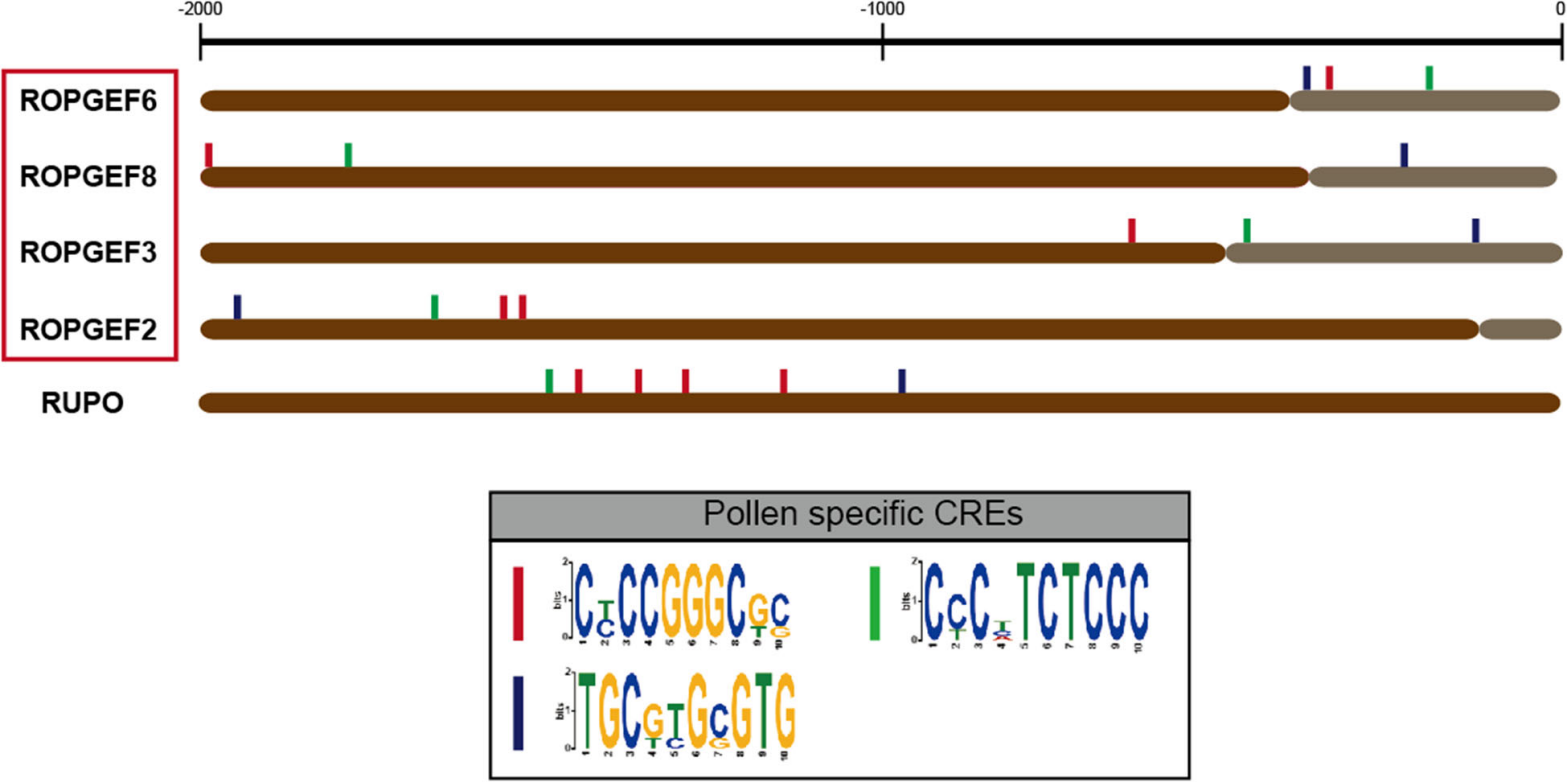
Promoter analysis of four pollen-specific-expressed *OsRopGEF* genes. Identification of putative *cis*-acting regulatory elements (CREs) specifically found in pollen-specific-expressed *OsRopGEF* genes and RUPO, using the MEME-suite. The number of scale bars above the figure shows the upstream position of the promoter base pair when taken at + 1 of ATG. We analyzed up to the upstream 2000-base pair. Gray bar indicated the 5`-untranslated regions and brown bars indicated the promoter regions

We then searched to find CREs that existed in the promoters of only pollen-preferred *OsRopGEF*s. Firstly, we searched for motifs that were common to the promoters of the four pollen-preferred genes, using MEME (Fig. 6). We downloaded the 2000-base pair upstream sequences of the four *OsRopGEF* genes and found ten conserved elements. Among them, three CREs were absent from the promoters of the other, pollen-non-preferred *OsRopGEF* genes. These three CREs were present at − 2000 to − 1500 bp from the transcription start sites of *OsRopGEF3* and OsRopGEF6, and mainly existed within − 1000 bp of the transcription start sites of *OsRopGEF2* and *OsRopGEF8.* All CREs were present once in the promoter of each gene, except for the first CRE (presented as a dark blue box; Fig. 6), which was present twice in the promoter of *OsRopGEF2*. We predicted that these three CREs will be involved in pollen expression, indicating their functional similarity in the transcriptional regulation process. We analyzed whether these three CREs are conserved in the promoter of *AtRopGEF*, which is highly expressed in pollen. Although *ATRopGEF2* has the first and the third CREs, and *ATRopGEF12* has the third CRE, the other pollen-expressed *AtRopGEF* genes do not contain, indicating that the different regulating system between rice and Arabidopsis might exist. However, *OsCrRLK1L13* (LOC_Os06g03610), also known as *RUPO* (*Rupture Pollen tube*), possesses these three CREs in promoter regions. RUPO is highly specifically expressed in pollen and is known to regulate pollen-tube growth and integrity in rice [36]. Our results indicate that these three CREs could be candidate for rice pollen-specific gene regulation, and further examination such as deletion assay remains for the clarification of our estimation. Next, using TOMTOM, we revealed that these elements were present in transcription factor genomic sequences such as MYB and bHLH, indicating that the four *OsRopGEFs* could be regulated in pollen by transcription factors such as MYB, and bHLH, for example.

### Plant phenotype of *RopGEF* mutant

**Fig. 7:**
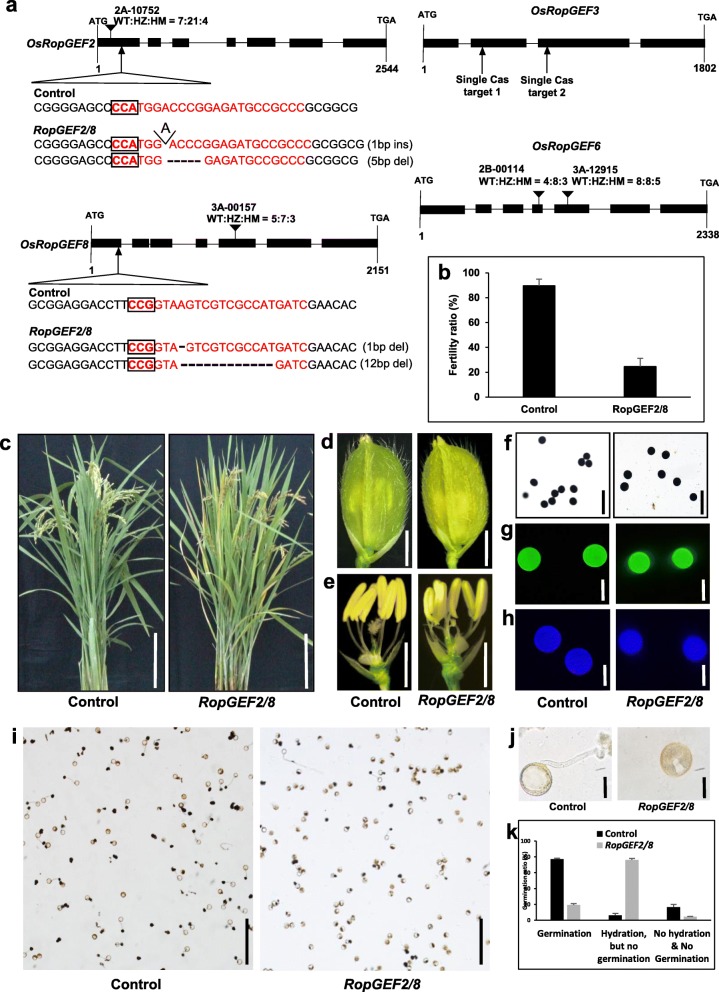
Identification of RopGEF mutants using T-DNA insertion and CRISPR-Cas9 system. **a** Scheme of the DNA structure and mutant regions. In the gene structure figures, the black boxes are exon region, black lines are intron region, and the arrow indicates the position of mutation occurs. Red letters indicate the target site of sgRNA, and black box indicate the PAM site in front of the sgRNA target sites. Ins; insertion, del; deletion. **b** Fertility ratio comparison between wild-type and *ropgef2/8* mutant plants. Error bars are standard deviation of more than three panicles in the plants. Phenotype of plant growth (**c**) Bar = 10 cm, and (**d**) flower structures, Bar = 2 mm, (**e**) reproductive organs formation, Bar = 2 mm, (**f**) pollen starch accumulation stained with KI, Bar = 20 μm, (**g**) pollen wall exine staining using auramin O, (**h**) pollen wall intine staining using calcoflour white, Bar = 20 μm, (**i**) In-vitro pollen germination test in solid pollen media, Bar = 250 μm, (**j**) enlarged pollen pictures, Bar = 10 μm, (**k**) Pollen germination ratio in vitro environment. Black bar indicates wild-type pollen; grey bar indicates RopGEF2/8 mutant pollen. Error bars are standard deviation of three technical repeats

To determine the function of RopGEF in rice pollen tube growth, we identified a T-DNA insertional lines at T2 generation: 2B-00114 and 3A-12,915, having T-DNA within *RopGEF6,* 3A-00157 and 2A-10,752, having T-DNA in *RopGEF8* and *RopGEF2*, respectively. Flaking region of about 10 kb T-DNA insertion was identified into exon region near to 5′ region of genes, indicating their functional gene loss. Therefore, we considered these T-DNA lines as knock-out lines and confirmed the seed segregation ratio. All the single T-DNA insertional lines exhibit normal growth and produce homozygous seed production (Fig. 7a), indicating the male gametic transmission is not disturbed by single gene knock-out, probably due to functional redundancy as shown in Arabidopsis [37]. To confirm it, we used CRISPR/Cas9 system to generate homozygous multiple knock-out mutants at T_0_ generation [38]. In the knock-out homozygous mutants of *RopGEF2*, the fertility ratio was slightly reduced but comparable to wild-type. Pollen germination and tube growth did not show difference compared with wild-type plants. In addition, single knock-out homozygous mutants of *RopGEF3* did not show any significant changes for growth and seed production, indicating that the male-gametic transmission is normal (Additional file 6).

In contrast, *RopGEF2* and *RopGEF8* double homozygous bi-allelic plants (*ropgef2/8*) (Fig. 7a), reduced seed fertility up to 20–30% compared with wild-type plants (Fig. 7b). There was no significant difference in vegetative and reproductive development between wild type and mutants (Fig. 7c-e). To investigate the possibility of any defect in pollen grain formation, mature pollen grains were examined. The starch formation of mature pollen grains assayed by iodine staining in double knock-out mutant did not differ from that of the wild type (Fig. 7f). To check whether pollen grain formed intact pollen walls, we also stained mature pollen grains with auramine O and calcofluor white and confirmed no differences between mutant and wild type (Fig. 7g-h). Next, we examined pollen germination behavior of double mutant pollens. Under in vitro pollen germination condition, about 77% wild-type pollen germinated among 83% hydrated pollen grains. In contrast, only about 20% of *ropgef2/8* double mutant pollens germinated, even though 75% of the mutant pollens were hydrated (Fig. 7i-k). These data indicate that *OsRopGEF* genes are functionally redundant for pollen germination in rice. Further different combination of multiple gene mutants could explain functional relationship between four pollen-specific RopGEF genes.

## Discussion

In animal and plant cells, a wide range of key cellular processes that require the establishment of cell polarity are governed by Rho GTPases or ROPs [6, 39, 40]. ROPs transduce intracellular and extracellular stimuli in a spatially and temporally regulated manner, resulting in localized regulation of intracellular responses [6]. The number of ROP proteins varies among plant species: seven in rice, nine in maize, and 11 in Arabidopsis. In Arabidopsis, three *ROPs* (*ROP1*, *ROP3*, and *ROP5*) are highly expressed in pollen and are functionally redundant in the regulation of pollen tube growth. AtROP1 is localized to the apical region of the pollen tube and is dynamically regulated by endocytosis during pollen tube growth [41, 42]. Disruption of *AtROP1* by overexpression of the constitutively active form resulted in swollen tubes whereas disturbance of AtROP1 activation resulted in short pollen tubes [43, 44], indicating that the control of ROP activity level is important for pollen tube shape. By contrast, there was no pollen-preferred *ROP* member in rice, as analyzed by the heatmap of tissue expression (Additional file 7a). Instead, *OsRac6*, a phylogenetically close member of *AtROP1/3/5* (Additional file 7b and c), was expressed in various tissues including mature anthers and pollen, suggesting that control of ROP activity through control of one member, *OsRac6,* might be important in rice pollen germination.

To achieve regulation of ROPs, plants might have evolved specific regulators such as RopGEFs [9]. As a result of comparative expression analysis, we found that four and five genes were highly expressed in pollen among 11 rice and 14 Arabidopsis *RopGEF* family members, respectively. This suggests that the ROP regulatory mechanism by RopGEF in pollen might be conserved between rice and Arabidopsis. Quadruple mutants of *AtRopGEF1, AtRopGEF9, AtRopGEF12,* and *AtRopGEF14* showed reduced pollen tube elongation, though this effect was not exhibited by any single gene mutation [16]. Due to functional redundancy, single mutants showed no defects in pollen germination and tube growth. Knock-out mutants of *OsRopGEF7B* were ubiquitously expressed in different tissues, including pollen, causing abnormal development of floral organs but not altered with respect to pollen formation or pollen germination [29]. Instead, *OsRopGEF2, OsRopGEF3, OsRopGEF6,* and *OsRopGEF8* were preferentially expressed in pollen, strongly suggesting their significant roles in pollen germination and tube growth. Our phenotype analysis revealed the defect of pollen germination in generated double homozygous mutant of *OsRopGEF2* and *OsRopGEF8*, reducing fertility ratio, while there is no significant defect on pollen germination in any single mutant. It suggests that rice pollen germination and tube growth require at least two RopGEF genes and there is functional redundancy among *RopGEF* genes, as in Arabidopsis.

The pollen-preferred expression pattern of the four genes might be explained by the existence of the three *cis*-acting elements absent from promoters of the other seven, pollen-non-preferred members. Since these *cis*-acting elements also exist in promoters of regulatory genes such as *MYB* and *bHLH*, we expect that either MYB or bHLH would be the potential transcription factors regulating the expression of the *RopGEF*s. However, results from the MEME-suite are required to demonstrate the binding site of the *cis*-acting elements in promoters of the transcription factors, through further analysis such as the use of the yeast-one hybrid system.

Despite of variable isoforms, gene sequences and protein structures are well conserved throughout AtRopGEFs and OsRopGEFs, especially with respect to the PRONE domain, indicating the conserved activity of the RopGEFs. The differences in the number of alpha helices in the RopGEF 3-D structure may be involved in stabilizing the WW-loop conformation [45]. The 3-D structures of OsRopGEF2 and 6 display the same length of the WW-loop (40 residues) compared with the reported AtRopGEF8 [32], although OsRopGEF3 and 8 exhibit shorter lengths, 34 and 25 residues, respectively (Table 1). The WW-loop is known to be variable in its length and sequence among different members of the RopGEF family [8, 9, 32, 45]. We found that the phosphorylatable serine residue [13] was also conserved in the pollen-preferred OsRopGEFs as in the AtRopGEFs. Further investigations will be required to determine how the four pollen- preferred OsRopGEFs are regulated by interacting partners such as the pollen-preferred RLKs [17, 46].

Our report of differential distribution of OsRopGEFs in tobacco epidermal cells indicates that they could regulate different processes of pollen germination. In Arabidopsis, it was observed that RopGEFs interacted differentially with the GTP- and GDP-bound forms of ROP1 and were localized in different subcellular locations [8]. Deletion of the C-terminal domain in AtRopGEF12 abolished the membrane association when it was expressed in tobacco pollen [8]. RopGEFs probably activate ROPs in the PM, and their spatial distribution reflects the sites of ROP activation [6]. It was suggested that the apical location of ROP1 in turn feed-forward regulates exocytosis of RLK and RopGEFs [5]. Another study reported that the phosphorylation of the PRONE domain of RopGEFs by AGC kinase was critical for the localization of RopGEFs to the apical PM, and subsequently for ROP activation and pollen tube polarity [47]. Cellular ROP signaling is also involved in root hair formation, another polar tip-growing plant cell type. Recently, functional analysis revealed that root-hair-preferred AtRopGEF3 functions in root hair initiation by AtROP2 polarization, while another one, AtRopGEF4, regulates subsequent root hair growth [48]. Therefore, it is possible that RopGEF members can be regulated differently by kinases to achieve different localizations and activations, and subsequently function in different phases of polar cell initiation and growth. Further studies of the dynamic distribution of OsRopGEF by other signals or interacting partners would provide a better understanding of the molecular network necessary for the process of rice pollen germination.

## Conclusions

Our study identified four genes that are highly expressed in pollen among RopGEF genes in rice, and confirmed their conserved domain in protein sequence, similarity in protein secondary and tertiary structure, and identified each subcellular localization. In addition, we confirm that double knock-out mutant of *RopGEF2* and *RopGEF8* significantly reduced pollen germination and seed production. We also found a novel cis-regulatory element that is expected to affect pollen expression by promoter analysis and present RopGEF model in the pollen cell. Our study could provide valuable information on the functional study of RopGEF in rice.

## Methods

### Multiple sequence alignment and phylogenetic tree construction

To perform a phylogenetic analysis of RopGEF and ROP/Rac in rice and *A. thaliana*, we collected the protein sequences with locus ID from the Rice Genome Annotation Project (http://rice.plantbiology.msu.edu/), the National Center for Biotechnology Information (NCBI, https://www.ncbi.nlm.nih.gov/) and the Phytozome platform (https://phytozome.jgi.doe.gov/pz/portal.html) (Additional file 8). Multiple amino acid sequences were aligned using ClustalW [49]. The phylogenetic analysis was performed using MEGA 7.0.26 under maximum likelihood and neighbor-joining methods [50].

### Meta-expression analysis

We used a publicly available rice Affymetrix microarray data set prepared from diverse tissues include anthers and pollen from the NCBI GEO [51] to identify late-pollen-preferred genes (GSE21494, GSE109811, [31]). To examine these data, we used the Affy package encoded by R language to normalize the signal intensity and then transformed them into log_2_ values. The normalized data, with averaged Affymetrix anatomical meta-expression data, were then used for further investigations, e.g., heatmap construction, and identification of the late-pollen-preferred genes [52].

### Plant growth and RNA extraction

Rice materials including cv. Dongjin wild type, and T-DNA insertion lines used in this study were obtained from T-DNA storage bank in Kyung Hee University (http://signal.salk.edu/RiceGE/RiceGE_Data_Source.html). To grow rice, the seeds were sterilized with 50% of sodium hypochlorite for 30 min, washed with distilled water, and then germinated on Murashige and Skoog (MS) media under controlled conditions in 7 days (28/25 °C day/night, 8-h photoperiod, and 78% relative humidity). The seedlings were grown in the greenhouse for 1 months and then transferred to a paddy field of Kyung Hee University. Plant cultivation and collection of plant materials were performed in accordance with permission and regulation on living modified organism guided by Korean government. For subcellular localization, tobacco (*Nicotiana benthamiana*) plants were grown as previously described [53] in the growth chamber.

For gene expression analysis, various rice tissues were separately frozen in liquid nitrogen and ground with a Tissue-Lyser II (Qjagen; Hilden, Germany). For pollen isolation, we collected mature pollen in an RNA stabilization solution (RNAlater Tissue Collection; Invitrogen) from dehiscent anther which just started to open. RNA was extracted with TRIzol buffer as follows [54], and cDNAs were synthesized using SuPrimeScript RT premix from GeNet Bio [55]. For tissue -specific expression by qPCR, we used control primer sets for rice ubiquitin 5 (*OsUbi5*, LOC_Os01g22490). All of the qRT-PCR primers we have used in our experiments are listed here (Additional file 9). The PCR cycle conditions used were 95 °C for 30 s, 57 °C for 30 s, and 72 °C for 1 min 30 s for 22–38 cycles. For real-time PCR (qPCR), we used cycling conditions of 95 °C for 15 s, 57 °C for 30 s, and 72 °C for 60 s using Roter-Gene Q instrument system (Qiagen, Hilden, Germany). We used 2x Prime Q-Mastermix (GeNet Bio) which contains SYBR Green1 for qPCR buffer. To determine the significant expression changes of four *RopGEF* genes in different tissues, we performed qPCR analysis with three independent biological replicates. Relative transcript levels and fold change were calculated by the 2^−*∆Ct*^ and 2^−∆∆*Ct*^ methods, respectively [56].

### Protein structure analysis

The three-dimensional (3-D) structural models were computed on a SWISS-MODEL Workspace in a fully automated mode [57] using RopGEF amino acid sequences as templates. The obtained 3-D structures were visualized using the University of California, San Francisco (UCSF) Chimera 1.10 program [58].

### Cis-acting elements analysis

To analyze the pollen-specific promoter regions of four *OsRopGEF* loci, we extracted 2-kb upstream sequences from the start codon of 11 *OsRopGEF* loci from EMBL (https://plants.ensembl.org/*Oryza*_*sativa*/Info/Index) and Gramene (http://ensembl.gramene.org/*Oryza*_indica/Info/Index). To find the *cis*-elements in the promoter sequences of the *OsRopGEF* genes, the 2-kb upstream sequence was scanned using the PLACE (Plant *cis*-acting regulatory DNA elements) database [59]. To discover novel motifs, which exist only in pollen-specific RopGEF genes, we performed Multiple Em for Motif Elicitation (MEME)-suite searches with those sequences in the FASTA format via the Web server hosted by the University of Queensland (http://meme-suite.org/tools/meme) [60]. First, we analyzed the 2-kb promoter sequence of all 11 *RopGEF* genes at once. Next, the 2-kb promoter sequences of the four pollen-specific RopGEF genes were analyzed, and we compared the two data sets using MEME. Among the motifs discovered, we chose those that were found only in the promoter sequence of pollen-specific *RopGEF* genes and searched for some motifs in databases of known motifs, using TOMTOM. The search conditions were “motif length 5 to 10bp,” “indicating only the motifs commonly found in the input data,” and “checking both strands.”

### Subcellular localization analysis

The coding sequences (CDSs) of four *RopGEF* genes were amplified from mature anther cDNA and cloned into pGreen vector fused with C-terminal green fluorescence protein (GFP). All of the cloning primers we have used in our experiments are listed here (Additional file 9). The constructs were transfected into *A. tumefaciens* strain GV3101 and used for *Nicotiana benthamiana* infiltration as described by [61]. Two to 3 days after infiltration, GFP fluorescence was observed with a confocal laser scanning microscope (Zeiss LSM 510, Jena, Germany) with spectral settings of 500–530 nm for emission and 488 nm for excitation. For staining tobacco leaves, the plasma and vacuolar membrane marker FM4–64 (Thermo Fisher Scientific) was used. We treated 0.1% FM4–64 to tobacco leaves for more than 15 min in dark condition and observed in red fluorescence protein (RFP) channel in 558 nm wavelength.

### Vector construction and rice transformation

To design guide RNA for single and multiple Cas vector cloning, we selected two target regions for each locus by CRISPRdirect tool [62]. For CRISPR-Cas9 vector cloning, we synthesized oligo dimers with annealed primers and ligated the dimers with the pRGEB32 binary vector [38]. Ligated vectors were transformed into *Escherichia coli,* and confirmed the insert by sequencing. After, the plasmid was transformed into the *Agrobacterium tumefaciens LBA4404*. The transgenic rice plants were generated through the stable transformation via *Agrobacterium*-mediated co-cultivation [63]. All of the cloning primers we have used in our experiments are listed in Additional file 9.

### Phenotype analysis

For testing phenotypes in mature pollen grains, the starch accumulation was stained with 1% solution of Iodine (*I*_2_) and potassium iodide (KI) for 10–20 min. The intine was stained with 0.1% Calcoflour white for 10 min and the exine was stained with 0.001% Auramine O for 10 min. KI-stained pollen grain was observed with bright field channel, Auramine O-stained pollen grain was observed with Fluorescein isothiocyanate (FITC) channels in 495 nm wavelength, and Calcoflour white-stained pollen was observed with Ultraviolet (UV) channels in 180 nm to 400 nm wavelength. To test in vitro pollen germination, fresh pollen grains were incubated onto a pollen germination medium consisting of 20% sucrose, 10% PEG, 3 mM calcium nitrate, 40 mg/L boric acid, 10 mg/L vitamin B1 and 1% agar. After incubation at 28 °C for 10–30 min, pollen tubes were observed with a SZX61 microscope (Olympus, Tokyo, Japan).

## Supplementary information

**Additional file 1: Figure S1.** Meta-expression analysis and genome-wide identification of *RopGEF* in rice. The expressions from various rice tissues were examined using Microarray database. Yellow color in the heatmap indicates high level of expression; dark blue, low level of expression. Numeric values indicate the average of the normalized log2 intensity of microarray data.

**Additional file 2: Figure S2.** Meta-expression analysis of entire AtRopGEF genes. The heatmap was prepared using the Genevestigator. We chose five representative tissues, including pollen. It revealed that five Arabidopsis RopGEFs were highly expressed in pollen. The dark red color of the heatmap indicated the highest expression; white color, lowest expression.

**Additional file 3: Figure S3.** Protein sequence alignment domain analysis and conserved phosphorylated amino acid residues of C-termini of RopGEF genes. Every OsRopGEF and AtRopGEF protein sequence was collected and aligned, followed by the PRONE domain (C1, C2, C3) and the WW-motif. At the end part of the sequence, we found some conserved regions. According to previous studies, S510 in the C-terminus of AtRopGEF12 is involved in the C-terminal inhibition of GEF activity. As in AtRopGEF12, the serine residue is conserved in each of the OsRopGEF2, OsRopGEF3, and OsRopGEF8 genes but not in OsRopGEF6. However, K (Lysine) can also be phosphorylated.

**Additional file 4: Figure S4.** Zoom-image of subcellular localization in the tobacco epidermal cells. It shows an enlarged portion of Fig. 5. The first panels in left side showed control and RopGEF proteins’ GFP signal, the second panels showed RFP signal stained with the membrane marker FM4–64, the third panels showed bright image, and the last panels on the right indicate merged images. In addition to the membrane signal, the large spot seen in the RFP channel is due to Chlorophyll’s auto fluorescence. a-d, Control (pGREEN-GFP); e-h, RopGEF 2; i-l, RopGEF3; m-p, RopGEF6; q-t, RopGEF8. Bars = 10 um.

**Additional file 5: Figure S5.** Promoter analysis of each *OsRopGEF* gene. Specific *cis*-acting element (CRE) known to affect expression in pollen were identified using PLACE database, namely three pollen-CREs and the TATA box. The number above the yellow bar shows the upstream position of the promoter base pair when taken at + 1 of ATG. We analyzed up to the upstream 2000-base pair. The *p*-values indicate how significantly the four genes exhibiting high expression in pollen differed from the other seven genes.

**Additional file 6: Figure S6.** Single mutant assay using CRISPR-Cas9 system. (a-b, d-e) In-vitro pollen germination test of wild-type (a, d), *ropgef2* (b, c), and *ropgef3* (e-f) single mutants on solid pollen media, Bar = 50 μm; (c,f) enlarged pollen pictures, Bar = 20 μm. Each control and mutant pollen in-vitro assays were performed in the same environment on the same day. (g) The ratio of pollen germination. Black bar indicates wild type; grey bar indicates *ropgef2*; white bar indicates *ropgef3* mutant pollen. Error bars are standard deviation of three technical repeats. (h) Fertility ratio comparison between wild-type and *ropgef2* single mutant plants. Error bars are standard deviation of more than three panicles in the plants.

**Additional file 7: Figure S7.** Meta-expression analysis and genome-wide identification of the seven OsRac and ten AtRop genes. (a) Heatmap expression analysis of *OsRac* gene. (b) Heatmap of *AtRop* using Genevestigator. (c) Phylogenetic tree constructs including every *OsRac* and *AtRop*.

**Additional file 8: Table S1.** Gene identification of RopGEF genes and RAC/ROP genes.

**Additional file 9: Table S2.** OsRopGEF isogene-specific primers for qPCR and cloning.

## Data Availability

Not applicable.

## Abbreviations

CRE: Cis-regulatory elements
CrRLK: Catharanthus receptor-like kinase
Dbl: Diffuse B-cell lymphoma
GP: Germinated pollen
GTP: Guanosine triphosphate
MP: Mature pollen
PM: Plasma membrane
PRK: Pollen receptor-like kinase
PRONE: Plant-specific ROP nucleotide exchanger
ROP: Rho-type GTPases of plants
RopGAP: GTPase-activator protein for ROP
RopGEF: Guanine nucleotide exchange factors for ROP
ROS: Reactive oxygen species
