# Supplementary material for: Genome-wide analysis of RopGEF gene family to identify genes contributing to pollen tube growth in rice (Oryza sativa)
Source: BMC Plant Biol. 2020 Mar 4;20:95. doi: 10.1186/s12870-020-2298-5 (PMC7057574; doi:10.1186/s12870-020-2298-5)
Supplement: Supplementary file 1 — Additional file 1: Figure S1. Meta-expression analysis and genome-wide identification of RopGEF in rice. The expressions from various rice tissues were examined using Microarray database. Yellow color in the heatmap indicates high level of expression; dark blue, low level of expression. Numeric values indicate the average of the normalized log2 intensity of microarray data. [file 12870_2020_2298_MOESM1_ESM.docx]

**Additional file 1: Figure S1**. Meta-expression analysis and genome-wide identification of *RopGEF* in rice. The expressions from various rice tissues were examined using Microarray database. Yellow color in the heatmap indicates high level of expression; dark blue, low level of expression. Numeric values indicate the average of the normalized log2 intensity of microarray data.
