# Supplementary material for: Genome-wide analysis of RopGEF gene family to identify genes contributing to pollen tube growth in rice (Oryza sativa)
Source: BMC Plant Biol. 2020 Mar 4;20:95. doi: 10.1186/s12870-020-2298-5 (PMC7057574; doi:10.1186/s12870-020-2298-5)
Supplement: Supplementary file 2 — Additional file 2: Figure S2. Meta-expression analysis of entire AtRopGEF genes. The heatmap was prepared using the Genevestigator. We chose five representative tissues, including pollen. It revealed that five Arabidopsis RopGEFs were highly expressed in pollen. The dark red color of the heatmap indicated the highest expression; white color, lowest expression. [file 12870_2020_2298_MOESM2_ESM.docx]

**Additional file 2: Figure S2**. Meta-expression analysis of entire *AtRopGEF* genes. The heatmap was prepared using the Genevestigator. We chose five representative tissues, including pollen. It revealed that five Arabidopsis *RopGEF*s were highly expressed in pollen. The dark red color of the heatmap indicated the highest expression; white color, lowest expression.
