# Supplementary material for: Genome-wide analysis of RopGEF gene family to identify genes contributing to pollen tube growth in rice (Oryza sativa)
Source: BMC Plant Biol. 2020 Mar 4;20:95. doi: 10.1186/s12870-020-2298-5 (PMC7057574; doi:10.1186/s12870-020-2298-5)
Supplement: Supplementary file 3 — Additional file 3: Figure S3. Protein sequence alignment domain analysis and conserved phosphorylated amino acid residues of C-termini of RopGEF genes. Every OsRopGEF and AtRopGEF protein sequence was collected and aligned, followed by the PRONE domain (C1, C2, C3) and the WW-motif. At the end part of the sequence, we found some conserved regions. According to previous studies, S510 in the C-terminus of AtRopGEF12 is involved in the C-terminal inhibition of GEF activity. As in AtRopGEF12, the serine residue is conserved in each of the OsRopGEF2, OsRopGEF3, and OsRopGEF8 genes but not in OsRopGEF6. However, K (Lysine) can also be phosphorylated. [file 12870_2020_2298_MOESM3_ESM.docx]

**Additional file 3: Figure S3**. Protein sequence alignment domain analysis and conserved phosphorylated amino acid residues of C-termini of *RopGEF* genes. Every OsRopGEF and AtRopGEF protein sequence was collected and aligned, followed by the PRONE domain (C1, C2, C3) and the WW-motif. At the end part of the sequence, we found some conserved regions. According to previous studies, S510 in the C-terminus of AtRopGEF12 is involved in the C-terminal inhibition of GEF activity. As in AtRopGEF12, the serine residue is conserved in each of the *OsRopGEF2*, *OsRopGEF3*, and *OsRopGEF8* genes but not in *OsRopGEF6*. However, K (Lysine) can also be phosphorylated.
