# Supplementary material for: Genome-wide analysis of RopGEF gene family to identify genes contributing to pollen tube growth in rice (Oryza sativa)
Source: BMC Plant Biol. 2020 Mar 4;20:95. doi: 10.1186/s12870-020-2298-5 (PMC7057574; doi:10.1186/s12870-020-2298-5)
Supplement: Supplementary file 4 — Additional file 4: Figure S4. Zoom-image of subcellular localization in the tobacco epidermal cells. It shows an enlarged portion of Fig. 5. The first panels in left side showed control and RopGEF proteins’ GFP signal, the second panels showed RFP signal stained with the membrane marker FM4–64, the third panels showed bright image, and the last panels on the right indicate merged images. In addition to the membrane signal, the large spot seen in the RFP channel is due to Chlorophyll’s auto fluorescence. a-d, Control (pGREEN-GFP); e-h, RopGEF 2; i-l, RopGEF3; m-p, RopGEF6; q-t, RopGEF8. Bars = 10 um. [file 12870_2020_2298_MOESM4_ESM.docx]

**Additional file 4: Figure S4**. Zoom-image of subcellular localization in the tobacco epidermal cells. It shows an enlarged portion of figure 5. The first panels in left side showed control and RopGEF proteins’ GFP signal, the second panels showed RFP signal stained with the membrane marker FM4-64, the third panels showed bright image, and the last panels on the right indicate merged images. In addition to the membrane signal, the large spot seen in the RFP channel is due to Chlorophyll's auto fluorescence. a-d, Control (pGREEN-GFP); e-h, RopGEF 2; i-l, RopGEF3; m-p, RopGEF6; q-t, RopGEF8. Bars = 10 um.
