# Supplementary material for: Genome-wide analysis of RopGEF gene family to identify genes contributing to pollen tube growth in rice (Oryza sativa)
Source: BMC Plant Biol. 2020 Mar 4;20:95. doi: 10.1186/s12870-020-2298-5 (PMC7057574; doi:10.1186/s12870-020-2298-5)
Supplement: Supplementary file 5 — Additional file 5: Figure S5. Promoter analysis of each OsRopGEF gene. Specific cis-acting element (CRE) known to affect expression in pollen were identified using PLACE database, namely three pollen-CREs and the TATA box. The number above the yellow bar shows the upstream position of the promoter base pair when taken at + 1 of ATG. We analyzed up to the upstream 2000-base pair. The p-values indicate how significantly the four genes exhibiting high expression in pollen differed from the other seven genes. [file 12870_2020_2298_MOESM5_ESM.docx]

**Additional file 5: Figure S5**. Promoter analysis of each *OsRopGEF* gene. Specific *cis*-acting element (CRE) known to affect expression in pollen were identified using PLACE database, namely three pollen-CREs and the TATA box. The number above the yellow bar shows the upstream position of the promoter base pair when taken at +1 of ATG. We analyzed up to the upstream 2,000-base pair. The p-values indicate how significantly the four genes exhibiting high expression in pollen differed from the other seven genes.
