# Supplementary material for: Genome-wide analysis of RopGEF gene family to identify genes contributing to pollen tube growth in rice (Oryza sativa)
Source: BMC Plant Biol. 2020 Mar 4;20:95. doi: 10.1186/s12870-020-2298-5 (PMC7057574; doi:10.1186/s12870-020-2298-5)
Supplement: Supplementary file 6 — Additional file 6: Figure S6. Single mutant assay using CRISPR-Cas9 system. (a-b, d-e) In-vitro pollen germination test of wild-type (a, d), ropgef2 (b, c), and ropgef3 (e-f) single mutants on solid pollen media, Bar = 50 μm; (c,f) enlarged pollen pictures, Bar = 20 μm. Each control and mutant pollen in-vitro assays were performed in the same environment on the same day. (g) The ratio of pollen germination. Black bar indicates wild type; grey bar indicates ropgef2; white bar indicates ropgef3 mutant pollen. Error bars are standard deviation of three technical repeats. (h) Fertility ratio comparison between wild-type and ropgef2 single mutant plants. Error bars are standard deviation of more than three panicles in the plants. [file 12870_2020_2298_MOESM6_ESM.docx]

**Additional file 6: Figure S6.** Single mutant assay using CRISPR-Cas9 system. (a-b, d-e) *In-vitro* pollen germination test of wild-type (a, d), *ropgef2* (b, c), and *ropgef3* (e-f) single mutants on solid pollen media, Bar = 50 µm; (c,f) enlarged pollen pictures, Bar = 20 µm. Each control and mutant pollen in-vitro assays were performed in the same environment on the same day. (g) The ratio of pollen germination. Black bar indicates wild type; grey bar indicates *ropgef2*; white bar indicates *ropgef3* mutant pollen. Error bars are standard deviation of three technical repeats. (h) Fertility ratio comparison between wild-type and *ropgef2* single mutant plants. Error bars are standard deviation of more than three panicles in the plants.
