# Supplementary material for: Genome-wide analysis of RopGEF gene family to identify genes contributing to pollen tube growth in rice (Oryza sativa)
Source: BMC Plant Biol. 2020 Mar 4;20:95. doi: 10.1186/s12870-020-2298-5 (PMC7057574; doi:10.1186/s12870-020-2298-5)
Supplement: Supplementary file 7 — Additional file 7: Figure S7. Meta-expression analysis and genome-wide identification of the seven OsRac and ten AtRop genes. (a) Heatmap expression analysis of OsRac gene. (b) Heatmap of AtRop using Genevestigator. (c) Phylogenetic tree constructs including every OsRac and AtRop. [file 12870_2020_2298_MOESM7_ESM.docx]

**Additional file 7: Figure S7**. Meta-expression analysis and genome-wide identification of the seven OsRac and ten AtRop genes. (a) Heatmap expression analysis of *OsRac* gene. (b) Heatmap of *AtRop* using Genevestigator. (c) Phylogenetic tree constructs including every *OsRac* and *AtRop*.
