# Supplementary material for: Genome-wide analysis of RopGEF gene family to identify genes contributing to pollen tube growth in rice (Oryza sativa)
Source: BMC Plant Biol. 2020 Mar 4;20:95. doi: 10.1186/s12870-020-2298-5 (PMC7057574; doi:10.1186/s12870-020-2298-5)
Supplement: Supplementary file 8 — Additional file 8: Table S1. Gene identification of RopGEF genes and RAC/ROP genes. [file 12870_2020_2298_MOESM8_ESM.docx]

**Additional file 8: Table S1**. Gene identification of RopGEF genes and RAC/ROP genes in rice and Arabidopsis.

| **Gene** | **Locus ID** | **NCBI Gene ID** | **Gene** | **Locus ID** | **NCBI Gene ID** | **Gene** | **Locus ID** | **NCBI Gene ID** |
| --- | --- | --- | --- | --- | --- | --- | --- | --- |
| *OsRopGEF1* | Loc_Os10g40270 | None | *AtRopGEF1* | AT4G38430 | 830000 | *AtROP1* | AT3G51300 | 824293 |
| *OsRopGEF2* | Loc_Os05g48640 | None | *AtRopGEF2* | AT1G01700 | 839250 | *AtROP2* | AT1G20090 | 838598 |
| *OsRopGEF3* | Loc_Os02g17240 | None | *AtRopGEF3* | AT4G00460 | 827967 | *AtROP3* | AT2G17800 | 816290 |
| *OsRopGEF4* | Loc_Os02g47420 | None | *AtRopGEF4* | AT2G45890 | 819197 | *AtROP4* | AT5G45970 | 834637 |
| *OsRopGEF5* | Loc_Os01g62990 | None | *AtRopGEF5* | AT5G05940 | 831858 | *AtROP5* | AT4G35950 | 829750 |
| *OsRopGEF6* | Loc_Os01g48410 | None | *AtRopGEF6* | AT3G55660 | 824732 | *AtROP6* | AT4G35020 | 829654 |
| *OsRopGEF7* | Loc_Os09g37270 | None | *AtRopGEF7* | AT5G02010 | 830000 | *AtROP7* | AT5G45970 | 834637 |
| *OsRopGEF8* | Loc_Os01g55520 | None | *AtRopGEF8* | AT3G24620 | 822058 | *AtROP8* | AT2G44690 | 819077 |
| *OsRopGEF9* | Loc_Os04g47170 | None | *AtRopGEF9* | AT4G13240 | 826941 | *AtROP9* | AT4G28950 | 829016 |
| *OsRopGEF10* | Loc_Os05g38000 | None | *AtRopGEF10* | AT5G19560 | 832076 | *AtROP10* | AT3G48040 | 823959 |
| *OsRopGEF11* | Loc_Os07g29780 | None | *AtRopGEF11* | AT1G52240 | 841654 | *AtROP11* | AT5G62880 | 836408 |
| *OsRac1* | Loc_Os01g12900 | 4325879 | *AtRopGEF12* | AT1G79860 | 844325 |  |  |  |
| *OsRac2* | Loc_Os02g02840 | 4328116 | *AtRopGEF13* | AT3G16130 | 820858 |  |  |  |
| *OsRac3* | Loc_Os02g20850 | 4329132 | *AtRopGEF14* | AT1G31650 | 840052 |  |  |  |
| *OsROP4* | Loc_Os02g50860 | 4330693 |  |  |  |  |  |  |
| *OsROP5* | Loc_Os02g58730 | 4331272 |  |  |  |  |  |  |
| *OsRacB* | Loc_Os05g43820 | 4339304 |  |  |  |  |  |  |
| *OsRacD* | Loc_Os06g12790 | 4340590 |  |  |  |  |  |  |
