# Supplementary material for: Genome-wide analysis of RopGEF gene family to identify genes contributing to pollen tube growth in rice (Oryza sativa)
Source: BMC Plant Biol. 2020 Mar 4;20:95. doi: 10.1186/s12870-020-2298-5 (PMC7057574; doi:10.1186/s12870-020-2298-5)
Supplement: Supplementary file 9 — Additional file 9: Table S2. OsRopGEF isogene-specific primers for qPCR and cloning. [file 12870_2020_2298_MOESM9_ESM.docx]

| **Additional file 9: Table S2**. *OsRopGEF* isogene-specific primers for qPCR and cloning analyses. | | |
| --- | --- | --- |
| **Genes** | **Sense Primers** | **Antisense Primers** |
| **qPCR** |  |  |
| *OsRopGEF2* | 5'-CATTGCTCAGTCAGCTCTTG-3' | 5'-TGAAGAACATCTTCGATTCG-3' |
| *OsRopGEF3* | 5'-TGACGAGGAAGTGGCTGCAG-3' | 5'-AGCGACTCGATGTACACCTC-3' |
| *OsRopGEF6* | 5'-TTCGGTATTCGGGGAGCAGC-3' | 5'-ATGCTGGTTCCATCCGTGGA-3' |
| *OsRopGEF8* | 5'-CAAGTCTTGGACAACGGCGT-3' | 5'-TTGAAGTTGTCCAGGTAATC-3' |
| **pGREEN cloning** | |  |
| *OsRopGEF2* | 5'-TCGACGGTATCGATAAGCTT  ATGGCGCGGCCGCTGCTGA-3' | 5'-CAGGAATTCGATATCAAGCTT  GTGTCTAGATATTGGGCT-3' |
| *OsRopGEF3* | 5'-TCGACGGTATCGATAAGCTT  ATGGTGCGGTTCCTCCGG-3' | 5'-CAGGAATTCGATATCAAGCTT  GTGGCGCGCGGAGGGGCT-3' |
| *OsRopGEF6* | 5'-TCGACGGTATCGATAAGCTT  ATGGTGCGGCGGCACCTG-3' | 5'-CAGGAATTCGATATCAAGCTTC  CTATGAGAAAAGCTTTTC-3' |
| *OsRopGEF8* | 5'-TCGACGGTATCGATAAGCTT  ATGGCAGCGAGCGGCGGT-3' | 5'-CAGGAATTCGATATCAAGCTT  ATGGCGACCTGGTGGGCTT-3' |
| **Single CRISPR-Cas cloning** | |  |
| *OsRopGEF3* (Target 1) | 5'-GGCAGTCGAATGCGATCACGAA CC-3' | 5'-AAACGGTTCGTGATCGCATTCG AC-3' |
| *OsRopGEF3* (Target 2) | 5'-GGCAAGCGAGTTCTGGTACGAG AA-3' | 5'-AAACTTCTCGTACCAGAACTC GCT-3' |
| **Multiple CRISPR-Cas cloning** | |  |
| *OsRopGEF2* | 5'- TAGGTCTCCTCTCCGGGTCCA  GTTTTAGAGCTAGAA-3' | 5'-CGGGTCTCACATTGAGCTCAA  TGCACCAGCCGGG-3' |
| *OsRopGEF6* | 5'-TAGGTCTCCAATGTCGCGCGC GTTTTAGAGCTAGAA-3' | 5'-CGGGTCTCATCGCCATGATCG  TGCACCAGCCGGG-3' |
| *OsRopGEF8* | 5'-TAGGTCTCCGCGACGACTTAC  GTTTTAGAGCTAGAA-3' | 5'-CGGGTCTCAGAGATGCCGCCC  TGCACCAGCCGGG-3' |
